# Internet of Things-Based Energy Consumption-Aware Framework Design for Smart Grid Environment

**DOI:** 10.3390/s26102989

**Published:** 2026-05-09

**Authors:** Mustafa Alper Çolak, Cüneyt Bayılmış

**Affiliations:** 1 Mitrona Elektronik ve Yazılım Teknolojileri A.Ş., Pendik, İstanbul 34906, Türkiye; 2 Computer and Information Engineering, Institute of Natural Sciences, Sakarya University, Sakarya 54050, Türkiye; 3 Department of Computer Engineering, Faculty of Computer and Information Sciences, Sakarya University, Sakarya 54050, Türkiye; cbayilmis@sakarya.edu.tr

**Keywords:** artificial intelligence of things, energy-aware, internet of things, smart grid

## Abstract

The widespread adoption of Internet of Things (IoT) technologies in smart grids enables fine-grained monitoring and control of energy systems. However, maintaining grid stability remains challenging when electricity production decreases unexpectedly due to fault-prone operating conditions at power generation units. This paper proposes an Artificial Intelligence of Things (AIoT)-based adaptive energy management framework that supports online adaptive demand-side control by detecting production drop anomalies and translating them into priority-aware load control actions. In practical energy systems, purely reactive strategies that trigger actions only after a demand violation may introduce temporary production–consumption imbalance and operational stress; therefore, the proposed framework targets preventive and data-driven intervention. Instead of relying on electricity production forecasting or static load shedding, the framework learns normal production behavior offline and identifies deviations using machine learning techniques. A fault modeling approach is used to generate scenario-based training data, and Logistic Regression (LR), Random Forest (RF), and Extreme Gradient Boosting (XGBoost) algorithms are employed for anomaly detection and control magnitude estimation. Following anomaly identification, IoT-enabled devices are selectively regulated based on device priority levels via an MQTT-based communication infrastructure. The framework is evaluated through simulations conducted on the CupCarbon platform under normal and production degradation scenarios. Results demonstrate that early anomaly detection alone is insufficient without accurate estimation of the required demand reduction and that the proposed approach enables effective demand-side control while preserving critical loads, thereby supporting resilient smart grid operation.

## 1. Introduction

Nowadays, IoT-based applications are increasingly being adopted in smart cities, smart grids, smart buildings, healthcare systems, and remote monitoring and control applications. By interconnecting heterogeneous sensing, communication, and actuation devices, IoT enables continuous monitoring, data exchange, and remote control across distributed environments. In general, IoT-based systems consist of smart objects with sensing and communication capabilities and cloud or centralized platforms where the collected data are stored, processed, and analyzed [1,2,3].

**Figure 1:**
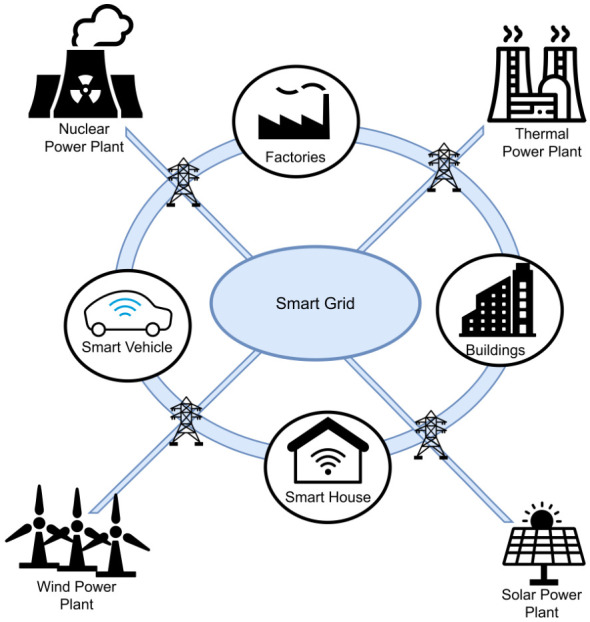
A typical smart grid environment.

A smart grid is an electrical power system enhanced with communication and information technologies to support bidirectional flows of electricity and information. As illustrated in Figure 1, smart grids integrate components such as smart meters, controllable appliances, distributed energy resources, and communication infrastructures. Their main objective is to maintain production–consumption balance and improve operational efficiency under dynamic operating conditions [4,5,6].

In recent years, smart grid management has become closely intertwined with IoT-based smart city applications. IoT technologies provide effective solutions for monitoring and managing energy consumption in smart grid environments by enabling continuous visibility of electricity usage and remote control of electrical devices. Recent studies further emphasize that the integration of IoT with artificial intelligence, intelligent analytics, and adaptive control mechanisms enables smart grids to evolve from passive monitoring infrastructures toward intelligent, autonomous, and adaptive energy management systems capable of operating under dynamic and uncertain operating conditions [7,8,9,10,11,12]. Such IoT-enabled infrastructures facilitate responsive and priority-aware demand-side interventions and improve the stability and reliability of energy management under abnormal system states.

Existing studies demonstrate the contributions of IoT technologies, advanced sensing, communication protocols, and distributed architectures to smart grid systems. Prior works have investigated smart sensors and metering infrastructures for energy monitoring, energy- and congestion-aware routing mechanisms for advanced metering networks, knowledge-based energy optimization strategies, IoT-enabled smart city architectures, energy-efficient communication protocols, and big data analytics infrastructures for smart grid sensor networks [6,13,14,15,16,17,18]. More recent studies have further explored AI-assisted monitoring, demand-side management, large-scale sensing, learning-based regional energy market mechanisms, microservices-based energy management, and adaptive control strategies in IoT-enabled smart grid and energy Internet environments [19,20,21,22,23,24,25].

Although several studies employ simulation platforms such as CupCarbon and integrate artificial intelligence techniques for energy optimization, demand-side management, or stability prediction, most existing approaches primarily focus on forecasting electricity consumption, optimizing load schedules, or detecting abnormal states as standalone outputs. In such studies, machine learning results are often used as auxiliary indicators rather than being directly translated into adaptive control decisions.

In contrast, the proposed framework explicitly targets the early identification of fault-prone operating conditions at power generation units and the estimation of the corrective control magnitude required before critical imbalance occurs. It links production-side anomaly detection with priority-aware demand-side control by using national-level continuously updated electricity production data and by translating detected production degradation into dynamic and selective load regulation actions. This distinction is particularly important under progressive and nonlinear production degradation scenarios, where early but inaccurate detection may still lead to insufficient or delayed intervention.

Maintaining the balance between electricity production and consumption becomes particularly challenging when generation levels suddenly decrease due to power plant outages, maintenance activities, or the intermittent nature of renewable energy sources. Conventional load shedding approaches and manual interventions often lack the flexibility required to respond effectively to such unexpected conditions. These limitations highlight the need for online, adaptive, and priority-aware demand-side intervention mechanisms that can operate reliably under fault-prone scenarios. Recent research has also highlighted the importance of data-driven, carbon-aware, and energy-aware AIoT frameworks to improve energy efficiency, operational sustainability, and adaptive decision making in large-scale distributed systems [26,27].

To clarify the novelty of the proposed framework, Table 1 compares representative recent AI-, IoT-, and AIoT-assisted smart grid studies with respect to anomaly/fault awareness, online/adaptive operation, demand-side control, priority-aware device-level regulation, and direct fault-to-control mapping.

As shown in Table 1, recent studies provide valuable contributions to smart grid stability prediction, production anomaly detection, adaptive resource allocation, and fog-based smart grid data analytics. However, these works generally focus either on detection and prediction tasks or on energy management and data processing mechanisms without directly coupling generation-side degradation awareness with priority-aware device-level demand-side control. In contrast, the proposed framework links production-drop anomaly detection with control-oriented energy saving estimation and distributed priority-aware load regulation, thereby establishing an explicit fault-to-control pathway within an online AIoT architecture.

In this context, this study proposes an AIoT-enabled adaptive energy management framework that detects abnormal operating conditions in power generation units using national-level streaming electricity production data and translates the detected production degradation into priority-aware demand-side control actions. The framework monitors generation information obtained from the EPİAŞ Transparency Platform and coordinates distributed IoT gateways through event-driven MQTT messages. In this structure, the central system computes the required energy saving ratio, while building-level gateways locally determine which non-critical loads should be temporarily regulated according to device priority classes and current consumption conditions. This structure establishes a direct pathway from production-side anomaly awareness to distributed, priority-aware demand-side regulation.

The main contributions of this study can be summarized as follows:

- An online AIoT-based adaptive demand-side energy management framework is proposed to support fault-aware and preventive production–consumption balancing under power generation degradation conditions.
- A production-side anomaly detection mechanism is developed using national-level electricity generation data and supervised machine learning models, namely LR, RF, and XGBoost, to identify fault-prone operating conditions.
- A control-oriented energy saving estimation approach is introduced to translate detected production degradation into a required demand reduction ratio, enabling anomaly detection outputs to be directly linked with demand-side control actions.
- A distributed and priority-aware load regulation strategy is designed using MQTT-based communication, where building-level IoT gateways locally determine device-level control actions according to device priority classes, current consumption profiles, and building-specific constraints.
- The proposed framework is evaluated through real data–driven CupCarbon simulations, and the performance of LR, RF, and XGBoost is comparatively analyzed using both classification metrics and control-oriented indicators.

The remainder of this paper is organized as follows. Section 2 presents the problem statement and motivates the need for fault-aware and adaptive demand-side control. Section 3 describes the proposed AIoT-based smart grid framework and its communication architecture. Section 4 introduces the intelligence and adaptive control mechanisms, including machine learning-based anomaly detection and priority-aware load regulation. Section 5 presents the simulation setup and performance evaluation. Finally, Section 6 concludes the paper and outlines future research directions.

## 2. Problem Statement

Modern power systems are undergoing a rapid transformation driven by the integration of renewable energy sources, the proliferation of IoT-enabled devices, and the increasing demand for online and adaptive energy management. In such smart grid environments, maintaining the dynamic balance between electricity production and consumption has become increasingly complex due to the variability of renewable generation, fluctuating demand patterns, and limited infrastructure flexibility. As a consequence, traditional demand-side management strategies struggle to provide timely and reliable responses to sudden production degradation events.

Despite significant advances in sensing technologies and wireless communication, existing energy management systems often exhibit several critical limitations:

- Lack of Fault-Aware Generation Monitoring:
  Many systems rely primarily on reactive control mechanisms and lack the ability to identify abnormal or fault-prone operating conditions at power generation units before production–consumption imbalance becomes critical.
- Insufficient Adaptivity in Demand-Side Control:
  Energy consumption is not managed in accordance with device criticality or building-specific contextual characteristics, resulting in inefficient and unnecessary load shedding that fails to reflect the actual severity and dynamics of generation disturbances.
- Inadequate Middleware Intelligence:
  Middleware components typically operate as passive data transmission layers, providing limited support for anomaly awareness, contextual reasoning, and adaptive decision-making processes.
- Scalability and Prioritization Challenges:
  Effectively managing communication and control across large-scale IoT deployments with heterogeneous devices and varying priority levels in diverse building environments remains a significant challenge.

These limitations lead to energy waste, unstable grid performance, and reduced user trust in smart grid solutions, particularly under anomalous or critical operating conditions. In such settings, purely reactive interventions that operate only after imbalance indicators emerge may introduce temporary instability; therefore, preventive fault awareness combined with accurate control magnitude estimation becomes essential for reliable grid operation.

To address these challenges, this study investigates and develops a comprehensive AIoT-based adaptive energy management framework. The considered system architecture incorporates:

- Machine learning–based power plant operational state classification using LR, RF, and XGBoost models,
- A rule-based anomaly confirmation mechanism to ensure reliable fault identification,
- A dynamic load control module that selectively deactivates electrical devices according to priority classes and building types,
- A lightweight MQTT-based middleware layer enabling intelligent communication, distributed control, and scalable topic orchestration.

By jointly addressing fault-aware monitoring and online adaptive demand-side control, the investigated framework aims to support operational continuity, optimize resource utilization, and enhance the operational resilience of future smart grid infrastructures.

## 3. Proposed Aiot-Based Smart Grid Framework

### 3.1. System Overview

This section presents an overview of the proposed AIoT-based smart grid framework designed to enable online, adaptive, and priority-aware demand-side energy management. The framework supports fault-aware production–consumption balancing by linking national-level electricity data with building-level IoT-enabled load control mechanisms. These national-level production data are obtained from the EPİAŞ Transparency Platform and serve as the primary input for fault awareness at the generation side.

The proposed framework follows a layered architecture consisting of four main functional layers: Data acquisition, middleware and communication, intelligence, and adaptive control. This modular design supports scalability and efficient coordination among heterogeneous smart grid components.

At the data acquisition layer, continuously updated electricity production and consumption data are collected from IoT-enabled generation units and buildings. Each building is equipped with a local IoT gateway that aggregates device-level measurements. In addition, temporal and environmental information is provided by a dedicated time–climate server to enhance contextual awareness.

The middleware and communication layer ensures bidirectional data exchange between distributed IoT devices, gateways, and the central control center using a lightweight publish/subscribe communication paradigm across both local area networks (LAN) and wide area networks (WAN).

The intelligence layer processes production, consumption, and contextual data to identify abnormal operating conditions and fault-prone states at power generation units and to estimate the severity of these conditions for control decision making. Based on these outputs, the adaptive control layer selectively and, when possible, preventively regulates IoT-enabled electrical devices according to predefined priority classes and building types, allowing critical loads to remain operational while non-essential loads are temporarily curtailed.

**Figure 2:**
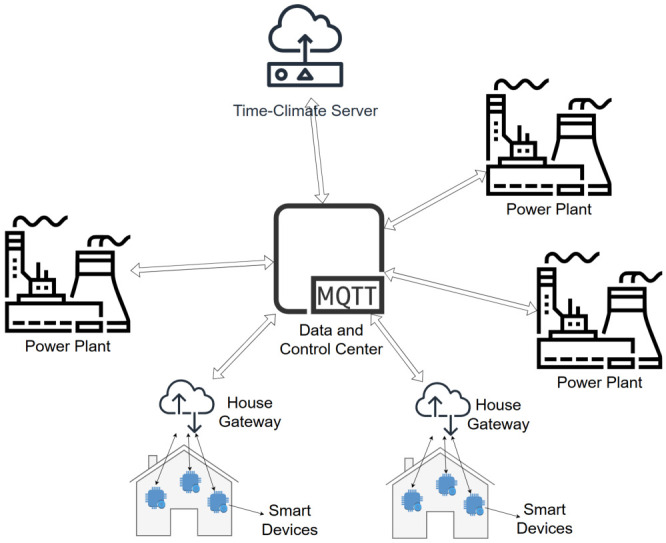
The proposed IoT-based smart grid framework architecture.

**Figure 3:**
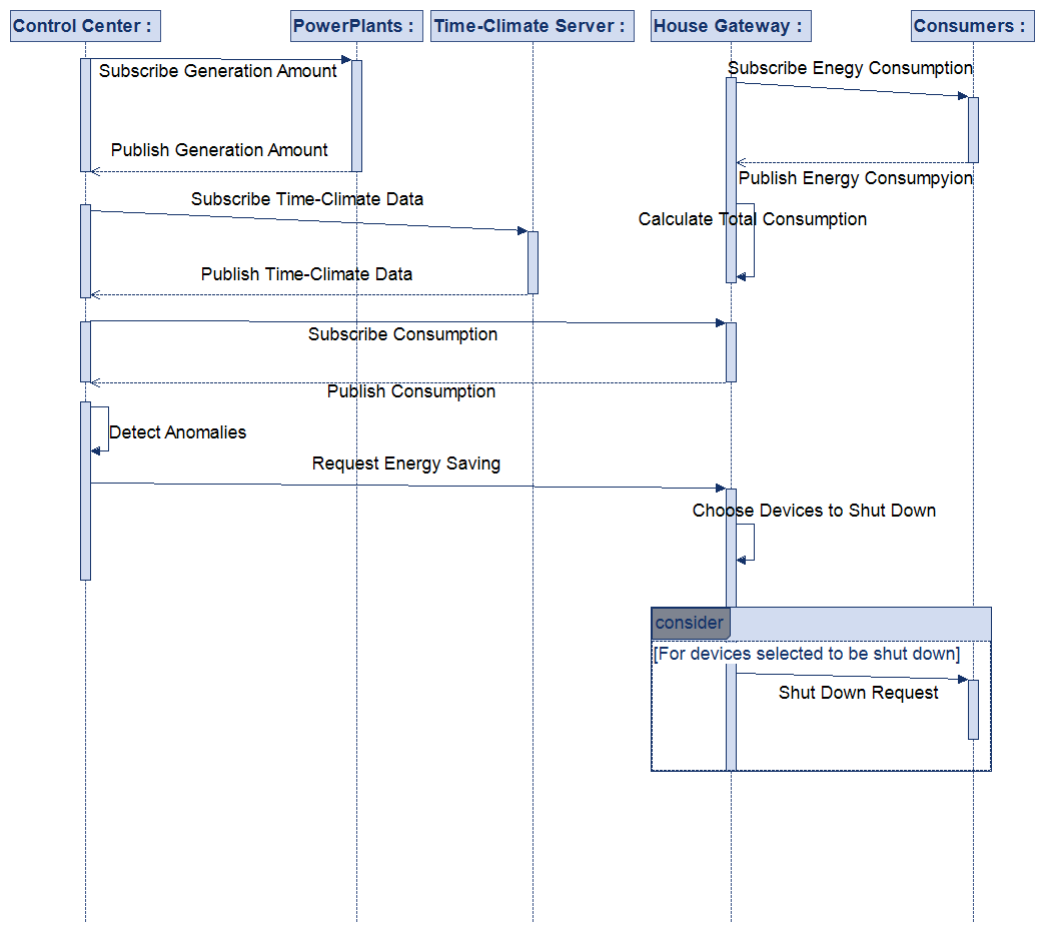
Sequence diagram for the algorithm.

Figure 2 illustrates the overall architecture of the proposed framework, highlighting the interaction between generation units, IoT-enabled buildings, middleware infrastructure, and the central intelligence and control components. Figure 3 presents the high-level sequence of operations, from data acquisition and data processing to anomaly detection and adaptive load control.

### 3.2. Deployment Architecture and Online Operation Assumptions

The proposed framework is designed for online adaptive demand-side energy management in smart grid environments. In this study, online operation refers to the continuous processing of streaming production and consumption data during system operation and the generation of adaptive demand-side control decisions according to the current grid state. The framework monitors production-side variations and demand-side consumption profiles, evaluates abnormal generation behavior, and updates the required energy saving ratio as new measurements become available. Thus, the proposed online operation enables adaptive demand-side regulation under evolving production degradation conditions without relying on offline batch analysis or static load-shedding rules.

The deployment architecture follows a hierarchical device–edge–fog/cloud structure. At the device layer, IoT-enabled sensors and actuators collect production and consumption measurements and execute local control commands. At the edge layer, building-level IoT gateways aggregate device-level consumption data, manage local device inventories, and apply priority-aware load control actions. At the fog/cloud layer, the central data and control center performs system-level data processing, machine learning inference, anomaly confirmation, and computation of the required global energy saving ratio.

In this architecture, machine learning models are trained offline using scenario-based production degradation data and executed online at the central data and control center. The central system does not directly determine which individual devices should be switched off or regulated. Instead, it computes the required energy saving ratio according to the detected production degradation and publishes this value to building-level gateways through MQTT topics. Each gateway then translates the received global energy saving ratio into local device-level actions based on its own device inventory, predefined priority classes, online consumption profile, and building-specific operational constraints.

This separation between fog/cloud-level intelligence and edge-level actuation reduces the communication and computational burden on the central system, improves scalability, and supports privacy preservation by avoiding the transmission of detailed device-level control decisions to the central layer. Moreover, since device-level regulation is performed locally by the gateways, the framework can adapt control actions to heterogeneous building conditions while maintaining a common system-level demand reduction objective.

### 3.3. Data Sources and Information

This section outlines the data sources used in the proposed framework and summarizes the information flow among system components. The proposed system integrates continuous, time-stamped energy data from both the production and consumption sides. Electricity production data are collected from IoT-enabled monitoring units deployed at power generation facilities, while consumption data are gathered from IoT-equipped electrical devices installed in residential, commercial, and industrial buildings.

Each building is equipped with a local IoT gateway that aggregates device-level measurements over a local area network and forwards the aggregated data to the central control center via a wide area network. This hierarchical communication structure supports scalable data collection and efficient information exchange.

In addition to production and consumption measurements, temporal and environmental context information is delivered to the central system to enhance situational awareness. All incoming data streams are synchronized and processed at the central control center to support subsequent anomaly detection and adaptive decision-making processes.

Overall, the information flow follows a closed-loop structure consisting of data acquisition, transmission, centralized analysis, and feedback-based control. This design enables timely detection of abnormal operating conditions and generation disruptions and supports responsive, priority-aware demand-side energy management.

### 3.4. Middleware and Communication Layer

The middleware layer plays a central role in the proposed smart grid framework by managing seamless communication between IoT-enabled devices, gateways, and the central management platform. This layer is implemented using the MQTT protocol, a lightweight and efficient publish/subscribe messaging system well-suited for constrained IoT environments and online energy data exchange. This selection is also supported by recent adaptive IoT protocol studies, where MQTT is highlighted as an effective option for lightweight and energy-aware IoT communication [32].

The smart grid system operates over two distinct network layers: LAN and WAN. Within each building, electrical devices are equipped with IoT sensor nodes connected via LAN to a local IoT gateway. These sensors continuously monitor energy consumption and transmit data to the gateway. The gateway aggregates, filters, and publishes consumption data to the WAN using MQTT topics.

On the generation side, each power generation facility is equipped with an IoT monitoring node that observes streaming operational signals and publishes this information over the WAN using predefined MQTT topics. These topics are dynamically structured according to generation type and criticality levels, enabling scalable and efficient topic hierarchy management without requiring manual reconfiguration.

The central data and control center subscribes to all MQTT topics published by both generation units and consumer gateways. It also receives temporal and environmental metadata (e.g., hour of day, temperature, humidity, solar irradiance) from a dedicated time–climate server. These contextual features are used to support fault-aware assessment and adaptive control mechanisms.

Upon the detection of abnormal operating conditions at the generation side, the control center does not directly determine which individual devices should be controlled or deactivated, avoiding centralized and rigid device-level decision making. Instead, it computes the required energy saving ratio needed to mitigate the anticipated production–consumption imbalance and broadcasts this value to building-level IoT gateways via MQTT topics.

Each building gateway autonomously translates the received energy saving ratio into concrete load control actions based on its local device inventory, predefined priority classifications, and building-specific operational constraints. This translation process is performed locally to reflect building-specific context and operational constraints. In this distributed decision-making approach, the control center provides a global adaptation objective, while the final device-level control logic is executed locally at the building level. This design significantly reduces central decision complexity, enhances scalability, and preserves user privacy by avoiding the transmission of detailed device-level information to the central system. Moreover, it enables flexible and context-aware demand-side management tailored to heterogeneous building environments under fault-prone operating conditions.

In summary, the middleware layer enables bi-directional, priority-aware, and fault-aware communication among all smart grid components. Through MQTT-based dynamic topic orchestration, it supports scalable data exchange, continuous monitoring, and adaptive demand-side control under abnormal operating conditions.

## 4. Intelligence and Adaptive Control

This section describes the intelligence and adaptive control mechanisms of the proposed AIoT-based smart grid framework. Unlike conventional approaches that focus on forecasting electricity production quantities, the proposed framework concentrates on identifying abnormal operating conditions and fault-prone states in power generation units and translating these conditions into priority-aware demand-side control actions, where accurate estimation of the required control magnitude is as important as early fault awareness. Accordingly, the effectiveness of the proposed framework is evaluated not only by detection timing but also by the stability and sufficiency of the resulting demand-side control actions.

**Figure 4:**
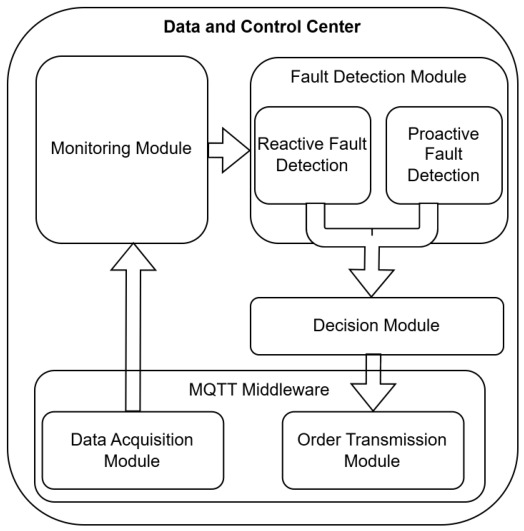
Internal structure of data and control center.

The intelligence layer combines machine learning–based operational state classification with rule-based anomaly confirmation to assess both the occurrence and severity of abnormal conditions, while the adaptive control layer executes selective and context-aware load control strategies at the consumer side. This hybrid design improves robustness by reducing spurious detections and ensuring that control actions are triggered only under confirmed fault-prone conditions. Figure 4 illustrates the internal architecture of the data and control center, highlighting the interaction between monitoring, fault detection, decision-making, and MQTT-based control execution modules that jointly support fault-aware and adaptive demand-side control.

### 4.1. Fault Modeling and Learning of Normal Operation

The first stage of the intelligence layer focuses on modeling fault scenarios and learning normal production behavior of power generation units. This stage represents an offline learning process, which establishes a behavioral reference space required for subsequent online anomaly detection and control-oriented decision making.

Instead of relying on historical fault records from real power plants, a fault model is defined to represent production degradation patterns that may indicate abnormal operating conditions. The fault model captures different types of production behavior, including gradual output degradation, sudden production drops, and irregular generation patterns observable at the sensor level. Using this model, a scenario-based and labeled dataset is generated, where each sample is associated with either normal or fault-prone operating conditions. This approach enables controlled and systematic exploration of fault dynamics that are difficult to observe comprehensively in real-world power plant datasets.

Two supervised machine learning algorithms, LR and RF, were initially trained on this dataset to recognize characteristic production behavior during normal operation. LR is used as a lightweight and interpretable baseline model, while RF is employed to capture nonlinear relationships and complex feature interactions present in diverse fault scenarios, thereby enriching the learned reference space.

The trained models do not directly trigger control actions at this stage; instead, they contribute to the construction of a reference representation of normal and fault-prone operational behavior that is later exploited by more advanced detection and control mechanisms during online operation, as described in the next subsection. In addition to these baseline learners, gradient boosting–based models are later employed to refine anomaly detection and, more importantly, control magnitude estimation under complex and evolving fault conditions.

### 4.2. ML-Based Proactive Fault Detection Framework

This subsection introduces the general machine learning-based framework used for proactive fault detection and early demand-side intervention. Rather than focusing on a specific classification model, the objective is to define a unified detection pipeline that can accommodate different learning algorithms and enable comparative performance evaluation. The term proactive is used to denote early-stage fault awareness and control preparation, rather than immediate device-level intervention.

The proposed framework operates by continuously monitoring electricity production and consumption data and constructing feature vectors that capture temporal trends, variability, and production–consumption margin dynamics. These features are then processed by supervised learning models trained offline to distinguish between normal and fault-prone operating conditions at power generation units.

Once an abnormal operating condition is detected, the system estimates the potential impact of the anticipated production degradation and computes the required energy saving ratio. This ratio is broadcast to building-level IoT gateways, enabling distributed and priority-aware demand-side control. In this study, LR, RF, and XGBoost classifiers are employed within the proposed framework, and their fault detection performance is comparatively evaluated under identical operating conditions.

The choice of supervised learning is motivated by the adopted scenario-driven fault labeling strategy. Although the EPİAŞ platform provides extensive real-world electricity production traces, explicit fault labels are not directly available in the raw production dataset. Therefore, normal and fault-prone operating samples were generated through the fault modeling procedure described in this study. This labeled structure enables supervised models to be trained and evaluated consistently. More importantly, the proposed framework requires a deterministic mapping between detected production degradation and the required demand-side control response. For this reason, supervised models were preferred over purely unsupervised anomaly scoring approaches in the present study.

These models were selected to represent different levels of model complexity and different trade-offs among interpretability, computational cost, inference latency, and predictive accuracy in the proposed online AIoT framework. LR is used as a lightweight and interpretable baseline model due to its low computational requirements and simple linear decision structure, which makes it suitable for fast online inference. However, its linear decision boundary limits its ability to capture nonlinear and progressively evolving production degradation patterns. RF is included as a nonlinear ensemble model capable of modeling feature interactions and improving robustness against noisy and heterogeneous fault patterns, at the cost of higher memory and computational requirements than LR. XGBoost is selected as a higher-capacity gradient-boosted tree model to improve predictive accuracy and control-oriented fault estimation under nonlinear and imbalanced production degradation scenarios. Although XGBoost introduces higher training complexity and inference overhead, this cost is acceptable in the proposed architecture because models are trained offline and online inference is performed at the central data and control center rather than on resource-constrained sensor nodes.

As summarized in Table 2, the selected models provide a structured comparison across three representative model families. LR provides a low-cost and interpretable baseline, RF offers a balance between nonlinear modeling capability and computational cost, and XGBoost prioritizes predictive capacity and control-oriented estimation accuracy. This comparison enables the evaluation of whether increased model complexity improves not only anomaly detection performance but also the quality of energy saving ratio estimation required for adaptive demand-side control.

Although unsupervised anomaly detection approaches, such as Isolation Forest, were considered in the early design phase, experimental observations indicated that these methods would only make a limited contribution to the scenario-driven, labelled fault modelling strategy adopted. In particular, the absence of explicit fault labels in unsupervised learning led to less stable fault-to-control associations and greater ambiguity when translating detected anomalies into deterministic demand-side control actions. Consequently, this study focuses on supervised classification models to ensure a reliable, interpretable and deterministic mapping of faults to controls within the proposed AIoT-based adaptive energy management framework.

### 4.3. LR–Based Fault Detection

In the first stage of the study, a LR model was employed to detect abnormal operating conditions in power generation units. LR was selected as an initial baseline classifier due to its low computational complexity, interpretability, and suitability for online decision-making.

Let x(t) denote the feature vector constructed at time step *t*, incorporating production levels, consumption statistics, and short- and long-term trend information. The LR model estimates the probability of a fault-prone operating condition as

(1) $$p_{f}\left(t\right)=\sigma \left(w^{⊤}x\left(t\right)+b\right),$$

where σ(·) is the sigmoid activation function, and w and *b* denote the model parameters learned during offline training.

A fault condition is declared when the estimated probability exceeds a predefined threshold. Upon detection, the predicted production degradation is used to compute the required energy saving ratio, which is then transmitted to building-level gateways to trigger adaptive demand-side control actions.

While LR provides fast and interpretable fault detection, its linear decision boundary limits its ability to capture complex nonlinear relationships present in diverse fault scenarios. This limitation motivated the exploration of more expressive models capable of providing not only earlier detection but also more accurate control magnitude estimation, as discussed in the next subsection.

### 4.4. RF–Based Fault Detection and Comparative Analysis

To address the limitations of linear classification observed in the LR–based fault detection stage, a RF classifier was subsequently employed within the same fault detection framework. The RF model was selected due to its ability to capture nonlinear feature interactions and its robustness against noise and heterogeneous fault patterns.

The RF classifier consists of an ensemble of *K* decision trees, where each tree independently maps the input feature vector $x\left(t\right)\in R^{d}$ to a binary fault decision,

(2) $$h_{k}\left(x\left(t\right)\right)\in \left\{0,1\right\},\;k=1,2,\ldots ,K.$$

The final fault probability is obtained by aggregating the outputs of individual trees using majority voting, which can be expressed in probabilistic form as

(3) $$p_{f}\left(t\right)=\frac{1}{K}\sum_{k=1}^{K}h_{k}\left(x\left(t\right)\right).$$

A fault-prone operating condition is declared when the aggregated fault probability exceeds a predefined threshold $\tau_{f}$. In contrast to LR, which assumes a linear decision boundary of the form $w^{⊤}x\left(t\right)+b=0$, the RF model implicitly constructs nonlinear decision regions through hierarchical feature partitioning and ensemble aggregation. This enables the detection of complex and gradual production degradation patterns that cannot be effectively captured by linear classifiers.

For a fair comparison, both LR and RF models were trained and evaluated using identical feature sets, fault scenarios, and simulation conditions. Performance was assessed using standard classification metrics, including accuracy, precision, recall, F1-score, and detection delay. Simulation results demonstrate that the RF model consistently detects production degradation earlier than the LR baseline and provides a more accurate estimation of the required energy saving ratio under non-stationary and noisy operating conditions.

Although the RF classifier significantly improves detection robustness compared to LR, its tree-level independence and majority voting mechanism limit its ability to fully exploit sequential error correction under progressively evolving fault scenarios. This observation motivates the investigation of more advanced ensemble learning techniques based on gradient boosting, as discussed in the following subsection.

### 4.5. XGBoost-Based Fault Detection

To address the limitations of tree-level independence and majority voting observed in the RF–based fault detection stage, an XGBoost classifier was employed. XGBoost is a tree-based ensemble learning method that constructs additive decision models through gradient boosting, enabling sequential error correction and more effective modeling of complex nonlinear feature interactions.

Given an input feature vector x(t), the XGBoost model estimates the fault likelihood by aggregating the outputs of multiple boosted decision trees:

(4) $$\hat{y}\left(t\right)=\sum_{k=1}^{K}f_{k}\left(x\left(t\right)\right),\;f_{k}\in F,$$

where F denotes the space of regression trees. The model parameters are optimized by minimizing a regularized objective function that balances prediction accuracy and model complexity.

Unlike RF, which aggregates independently trained trees, XGBoost optimizes each subsequent tree based on the residual errors of previous trees through gradient-based loss minimization. This sequential optimization mechanism allows the model to adapt more effectively to subtle and progressively evolving fault patterns, making XGBoost particularly suitable for capturing complex degradation dynamics observed in real-world power generation data.

As a consequence of this sequential learning strategy, XGBoost may exhibit a longer reactive detection delay compared to simpler models; however, it provides more reliable and sufficient control magnitude estimation, enabling stable and preventive demand-side operation.

### 4.6. Anomaly Detection Mechanism

This subsection describes the online anomaly detection mechanism employed during the runtime operation of the proposed framework. Unlike the offline learning stage, where machine learning models are trained using scenario-based fault data, the anomaly detection mechanism focuses on the continuous evaluation of incoming production and consumption measurements.

At runtime, streaming electricity production data are continuously collected from IoT-enabled monitoring units deployed at power generation facilities. These measurements are combined with consumption statistics and temporal features to construct a feature vector x(t) at each time step. The feature extraction process follows the same structure used during offline training to ensure consistency between training and inference phases.

Anomaly detection is performed using a selected supervised learning model trained offline, namely LR, RF, or XGBoost, without introducing additional hybrid or ensemble decision logic. The trained model processes x(t) and produces a fault likelihood or classification output that reflects the deviation of the current operating condition from learned normal production behavior.

To enhance robustness against transient fluctuations and measurement noise, anomaly decisions are not triggered by isolated model outputs. Instead, a sustained deviation criterion is applied, whereby an abnormal operating condition is confirmed only when consecutive fault indications persist over a predefined temporal window. This mechanism prevents unnecessary control actions caused by short-term variability or benign operational changes.

Once an anomaly is confirmed, it is represented as a system-level event and forwarded to the adaptive control layer. At this stage, no device-level control decisions are made. The anomaly detection mechanism solely signals the presence of a fault-prone operating condition and provides the necessary inputs for subsequent energy saving ratio computation and distributed demand-side control actions.

By decoupling online anomaly detection from device-level control logic, the proposed framework ensures modularity, scalability, and algorithm-independent operation. This design allows different machine learning models to be evaluated and compared within the same detection pipeline, while preserving a consistent and reliable operational workflow.

### 4.7. Priority-Aware Adaptive Load Control

Following the confirmation of an abnormal operating condition by the anomaly detection mechanism, the proposed framework activates a priority-aware adaptive load control process. Unlike centralized load shedding approaches, the control logic is deliberately distributed between the central control center and building-level IoT gateways to ensure scalability, flexibility, and privacy preservation.

Upon anomaly confirmation, the control center computes the required energy saving ratio ρ(t) based on the predicted production–consumption imbalance. This ratio represents the global demand reduction objective and is broadcast to all building gateways via MQTT topics. No device-level control decisions are made at the central level.

Each building gateway independently translates the received energy saving ratio into local control actions using its current consumption profile and predefined device priority classifications. Let $C_{b}\left(t\right)$ denote the total electricity consumption of building *b* at time *t*, and let $S_{b}\left(t\right)$ represent the set of devices selected for temporary regulation within that building. The local control objective is defined as

(5) $$\sum_{j\in S_{b}\left(t\right)}C_{j}\left(t\right)\geq \rho \left(t\right)\cdot C_{b}\left(t\right),$$

where $C_{j}\left(t\right)$ denotes the consumption of device *j*. Device selection follows a hierarchical priority policy, where critical devices are excluded from $S_{b}\left(t\right)$, and lower-priority devices are progressively considered until the required reduction target is satisfied.

In the current implementation, device priority classes are initialized using predefined criticality levels to ensure deterministic and safe load regulation. These priority levels are not imposed by the central control center; instead, they can be defined by building managers, facility operators, or authorized users according to building-specific operational requirements and user preferences. The role of the central control center is limited to detecting production-side abnormalities and computing the required global energy saving ratio. The translation of this ratio into device-level control actions is performed locally by each building gateway according to its own priority policy, current consumption profile, and operational constraints.

This separation of responsibilities allows the framework to support dynamic priority adaptation without embedding building-specific business rules in the central system. For example, local priority policies can be updated according to occupancy status, time of day, user preferences, seasonal requirements, or scenario-specific constraints. Critical loads remain protected as safety constraints, while the effective control order of non-critical loads can be adapted locally under changing operating conditions.

Load regulation is applied in a gradual and incremental manner to avoid abrupt service disruptions. Building gateways continuously monitor the effectiveness of applied control actions and adjust the selected device set in response to changing consumption patterns. This adaptive strategy ensures proportional load reduction aligned with the severity of the detected anomaly.

To maintain operational continuity and user trust, a recovery mechanism is incorporated into the control process. Once the anomaly detection mechanism indicates a return to normal operating conditions, previously regulated devices are automatically restored according to their priority levels. All control actions are logged with timestamps and contextual information to support traceability and system auditing.

By decoupling global adaptation objectives from local device-level decision making, the proposed priority-aware adaptive load control mechanism enables resilient, scalable, and context-aware demand-side management in AIoT-enabled smart grid environments.

### 4.8. End-to-End Fault-to-Control Workflow

This subsection summarizes the complete end-to-end workflow of the proposed AIoT-based smart grid framework, linking fault-prone operating condition detection at the generation side with distributed and priority-aware demand-side control actions. The overall operational flow of the system is illustrated in Figure 3, which depicts the sequence of interactions between generation units, the control center, and building-level IoT gateways.

The workflow begins with the continuous acquisition of electricity production data from IoT-enabled monitoring units deployed at power generation facilities, along with aggregated consumption measurements collected from building-level IoT gateways. These data streams are synchronized and processed to extract temporal, statistical, and margin-related features consistent with the offline training phase.

During online operation, the extracted feature vectors are evaluated by a selected supervised machine learning model trained offline to detect deviations from normal production behavior. This anomaly detection stage operates independently of the specific classification algorithm employed, enabling different models to be evaluated within the same detection pipeline. Once a fault-prone operating condition is confirmed, the system transitions from monitoring to control preparation without immediately enforcing device-level actions.

Following anomaly confirmation, the control center estimates the potential production–consumption imbalance and computes the required global energy saving ratio. This ratio represents a system-level adaptation objective rather than a device-level command and is disseminated to building gateways via MQTT-based publish/subscribe communication mechanisms.

At the building level, each IoT gateway independently translates the received energy saving ratio into local control actions based on continuously updated consumption measurements, predefined device priority classes, and building-specific operational constraints. Load regulation is applied incrementally, ensuring that critical devices remain operational while non-essential loads are selectively reduced to satisfy the required demand reduction target.

Throughout the control process, feedback from IoT-enabled devices and gateways is continuously monitored to assess the effectiveness of applied interventions. When production behavior returns to normal operating conditions, a recovery mechanism is triggered to restore previously regulated devices in accordance with their priority levels. All detection and control actions are logged with temporal and contextual information to support traceability and system-level auditing.

By decoupling anomaly detection, global adaptation objectives, and local device-level control decisions, the proposed end-to-end fault-to-control workflow achieves modularity, scalability, and resilience. This closed-loop design enables timely and proportional demand-side responses to production degradation while preserving user trust and operational continuity in AIoT-enabled smart grid environments.

## 5. Simulation and Performance Evaluation

### 5.1. Simulation Environment and Setup

To evaluate the effectiveness of the proposed AIoT-based adaptive energy management framework under realistic operating conditions, a real data–driven simulation methodology was adopted. Simulation experiments were conducted using the CupCarbon platform, which enables detailed modeling of smart grid components, IoT-enabled devices, communication infrastructures, and control interactions [33,34].

**Figure 5:**
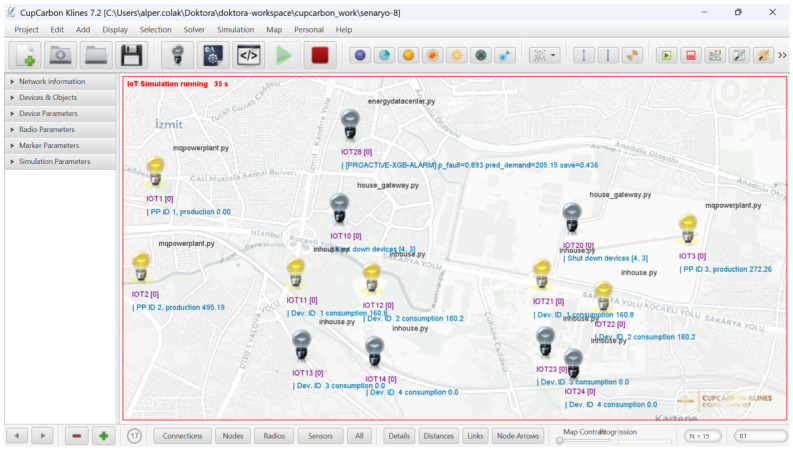
Simulation environment in CupCarbon.

The simulated environment represents a heterogeneous smart grid scenario comprising multiple buildings. On the supply side, distributed power generation units equipped with IoT-based monitoring capabilities were modeled to emulate real-world electricity production behavior. The whole simulation environment in CupCarbon is given in Figure 5, and key simulation parameters are given in Table 3.

To enhance the realism of the simulation and avoid reliance solely on synthetic data, real-world electricity production measurements were incorporated into the simulation framework. Specifically, electricity generation data were obtained from the EPİAŞ Transparency Platform, covering the Bursa, Cengiz, and Kazan natural gas combined cycle power plants. These data traces reflect actual national-scale production dynamics and were used to drive the generation profiles of the simulated power plants.

**Figure 6:**
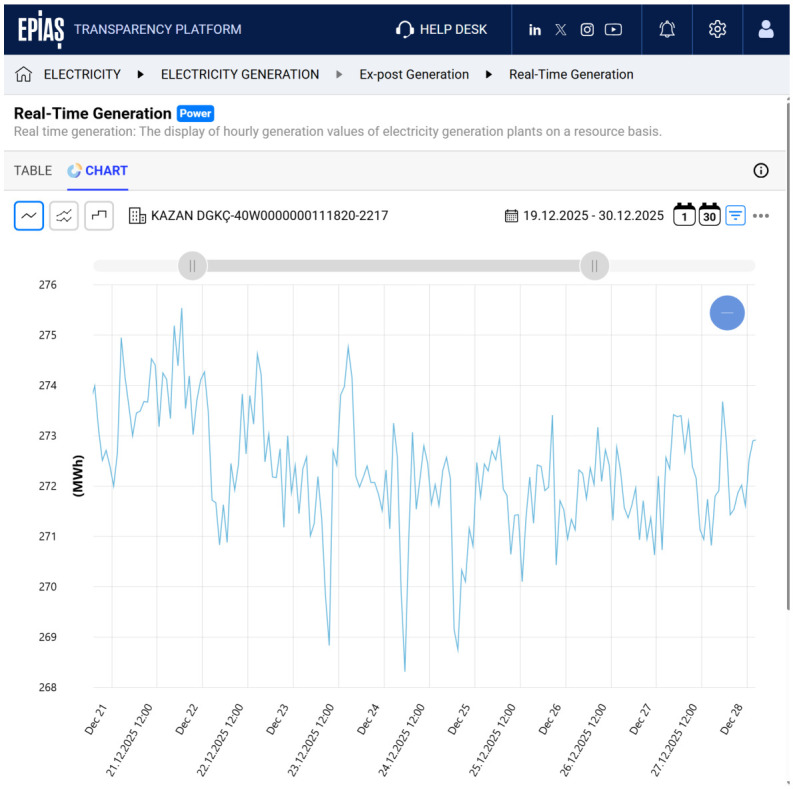
Production profile of Kazan Power Plant.

Electricity production and consumption data at both national and power-plant levels are provided by the EPİAŞ Transparency Platform, which serves as the official public data source of the Turkish electricity market [35]. The platform enables continuous monitoring of current and historical generation statistics for individual power plants as well as aggregated national production and consumption values. These data can be accessed through a web-based interface for visual inspection or programmatically via a RESTful API, allowing automated data retrieval for large-scale analysis and simulation studies. The EPİAŞ Transparency Platform is publicly accessible through its official web portal at https://seffaflik.epias.com.tr/, providing open access to electricity market data for both visual inspection and automated data retrieval. An example of time-stamped electricity generation data obtained from the platform is illustrated in Figure 6, which shows the hourly production profile of the Kazan Natural Gas Combined Cycle Power Plant. Such plant-level production traces were directly used to drive the generation profiles in the simulation environment.

In this study, the analysis focuses exclusively on natural gas combined cycle (NGCC) power plants. This choice is motivated by the relatively stable and controllable production characteristics of natural gas–fired units compared to renewable energy sources. Hydroelectric power plants exhibit significant output variability depending on water inflow and reservoir conditions, while wind power plants are inherently subject to rapid fluctuations driven by wind speed and meteorological conditions. These natural variations can obscure the distinction between normal operational behavior and fault-induced production degradation.

By contrast, natural gas combined cycle plants typically operate with smoother generation profiles and faster controllability, enabling a clearer separation between normal production dynamics and abnormal degradation patterns. This property makes NGCC plants particularly suitable for modeling progressive fault scenarios and for evaluating anomaly detection and adaptive demand-side control mechanisms without confounding effects caused by renewable generation variability.

The real production data were preprocessed and synchronized with the simulation timeline to generate realistic operating scenarios, including normal operation periods and production degradation events. Production drop scenarios were constructed directly from observed variations in the real data, enabling the evaluation of anomaly detection and adaptive load control mechanisms under conditions that closely resemble real grid behavior.

On the demand side, each simulated building was equipped with a local IoT gateway responsible for aggregating device-level consumption data and communicating with the central control center via MQTT-based publish/subscribe messaging. Device-level energy consumption was modeled according to priority classes ranging from critical to low, allowing realistic assessment of priority-aware demand-side control strategies.

Overall, the adopted real data–driven simulation setup enables comprehensive performance evaluation of the proposed framework under realistic generation conditions, while maintaining full control over system parameters and fault scenarios. This approach bridges the gap between purely synthetic simulations and real-world deployments, providing strong evidence of the practical applicability of the proposed solution [36]. From a deployment perspective, the proposed framework is compatible with a practical hierarchical smart grid architecture in which production-side measurements are collected from plant monitoring systems or market data interfaces, while building-level gateways execute local demand-side control actions. The central data and control center performs data aggregation, machine learning inference, anomaly confirmation, and computation of the required energy saving ratio. Since the final device-level decisions are made locally by building gateways, the framework does not require continuous centralized control of individual appliances. This design reduces communication overhead, improves scalability, and makes the proposed approach suitable for gradual deployment in existing IoT-enabled building and smart grid infrastructures. Although full field deployment is beyond the scope of the present study, the use of real production traces, MQTT-based communication, and gateway-level actuation logic provides a realistic basis for evaluating the feasibility of the proposed AIoT framework.

To improve the reproducibility of the proposed framework, the implementation details of the machine learning pipeline are also specified. The machine learning models were trained offline and then used for online inference within the CupCarbon/MQTT-based AIoT simulation. During online operation, the energy data center constructs the current feature vector from production and consumption measurements and evaluates the trained model whenever the system state is updated. The input data were organized as time-aligned production and consumption records. Missing values were handled by forward filling in order to preserve temporal continuity in the feature vectors during online inference. Feature engineering focused on dynamic production degradation indicators, including changes in production, changes in the production–consumption margin, short- and long-window variability measures, rolling slopes, and normalized standard deviation ratios. These features were selected to capture both rapid variations and gradual degradation trends in the production signal. The same feature extraction logic was used during offline training and online inference to ensure consistency between the training and deployment phases.

The LR model was implemented as a StandardScaler followed by elastic-net regularized Logistic Regression. The main parameters were penalty = elasticnet, solver = saga, l1_ratio = 0.5, C = 0.5, class_weight = balanced, max_iter = 5000, random_state = 42, and n_jobs = 1. The class_weight = balanced setting was used to reduce the effect of class imbalance between normal and fault-prone samples.

The RF model was configured as a nonlinear ensemble classifier with n_estimators = 800, max_features = sqrt, min_samples_leaf = 2, class_weight = {0:1,1:25}, random_state = 42, and n_jobs = 1. The increased number of trees and class weighting were used to improve robustness under imbalanced fault detection conditions while preserving deterministic reproducibility through the fixed random seed.

The XGBoost model used 1500 estimators, a maximum tree depth of 4, a learning rate of 0.03, subsampling and column-sampling ratios of 0.9, the binary:logistic objective, the logloss evaluation metric, reg_lambda = 1.0, reg_alpha = 0.0, scale_pos_weight = 70.0, early_stopping_rounds = 50, random_state = 42, and n_jobs = 1. The learning rate and regularization parameters were selected to improve training stability, while scale_pos_weight was used to compensate for the imbalanced distribution of fault-prone samples. Early stopping was applied to reduce the risk of overfitting.

For model validation, the faulty data were divided into training and test subsets using a stratified 75/25 split with random_state = 42, while no-fault data were used to characterize normal operating behavior. The fault labeling strategy was designed to support preventive control: samples preceding the first explicit energy-demand violation were treated as fault-prone according to the predefined preventive labeling window. Operational decision thresholds were selected using a threshold-sweep procedure on the test results, with the objective of reducing false positives while maintaining fault detection sensitivity.

The implementation environment was Python 3.12.8 with NumPy 2.2.1, pandas 2.2.3, scikit-learn 1.7.0, XGBoost 3.1.3, joblib 1.5.1, and paho-mqtt 2.1.0. The simulations were executed using CupCarbon KLINES 7.2 on a workstation equipped with an Intel Core i7-1355U processor and 32 GB RAM. All models were serialized using joblib after offline training, and online inference was executed inside the CupCarbon simulation through Python scripts communicating over local MQTT topics.

The performance of the proposed AIoT-based adaptive energy management framework is evaluated under three representative operational scenarios designed to assess anomaly detection reliability and adaptive load control effectiveness under both normal and fault-prone operating conditions.

Normal Operation Scenario: In this scenario, electricity production remains stable and no fault conditions occur at the generation units. This scenario is used to evaluate the ability of the proposed framework to correctly identify normal operating conditions, avoid false anomaly detections, and prevent unnecessary demand-side control actions. Production and consumption patterns follow nominal trends derived from real production data without artificial degradation.

Production Drop Scenario: In this scenario, one power generation unit experiences a progressive degradation in production output, emulating fault-prone operating conditions. This scenario evaluates the system’s ability to detect production drop anomalies during online operation and to initiate appropriate adaptive demand-side control actions.

Production degradation is modeled using a multi-phase fault evolution approach that reflects realistic power plant failure behavior. Specifically, the Bursa natural gas combined cycle power plant is subjected to a three-phase fault model consisting of residual drift, variance growth, and intermittent production drops.

Let P(t) denote the actual power output of the generation unit at time *t*, and let $\hat{P}\left(t\right)$ represent the expected production under normal operating conditions. The residual signal is defined as

(6) $$r\left(t\right)=P\left(t\right)-\hat{P}\left(t\right).$$

During the early warning phase (Phase 1), incipient fault behavior is modeled by introducing a gradual negative bias drift in the residual signal:

(7) r(t)=r(t−1)−δ,δ>0,

which results in a slow but persistent deviation from nominal production levels.

In the intermediate phase (Phase 2), increased operational instability is represented by a progressive rise in the variability of the residual signal. This behavior is quantified using a rolling standard deviation computed over a sliding window of length *W*:

(8) $$\sigma_{r}\left(t\right)=\mathrm{std}\left(r(t-W+1),\ldots ,r(t)\right),$$

where $\sigma_{r}\left(t\right)$ increases as the fault condition evolves.

In the final phase (Phase 3), the fault manifests as intermittent short-term production dropouts followed by a sustained production reduction or trip event. This behavior is modeled by introducing stochastic negative impulses into the production signal:

(9) $$P\left(t\right)=\left\{\begin{array}{cc} \gamma P(t), & \mathrm{with}\;\mathrm{probability}\;p_{d},\\ P(t), & \mathrm{otherwise}, \end{array}\right.\;0<\gamma <1,$$

where $p_{d}$ denotes the probability of a production dropout and γ represents the severity of the drop.

This three-phase fault modeling approach enables the simulation of realistic degradation trajectories derived from real production data and allows the proposed anomaly detection and adaptive load control mechanisms to be evaluated under progressively worsening operating conditions.

Comparative Model Evaluation Scenario: In this scenario, the anomaly detection performance of LR, RF, and XGBoost models is evaluated and compared under identical production drop conditions. All three models are trained using the same feature sets and fault scenarios and are tested against the same degradation trajectories applied to the Bursa power plant. Performance is assessed using standard classification metrics and detection delay to provide a fair and consistent comparison between all three approaches.

### 5.2. Results

The performance of the proposed AIoT-based adaptive energy management framework was evaluated under both normal operation and production drop scenarios. The evaluation focuses on anomaly detection reliability, adaptive demand-side control effectiveness, and the ability of the system to maintain production–consumption balance under progressive fault conditions.

A simple control strategy based on triggering corrective actions only when the energy demand exceeds zero was initially considered. In real power systems, such delayed interventions may also activate protection mechanisms and exacerbate cascading failures. However, such a reactive approach inherently allows temporary production–consumption imbalance, which may introduce system stress, instability, and protection-level faults in real-world energy systems. Consequently, this strategy was deemed unsuitable for practical deployment and was not adopted in the proposed framework.

Instead, machine learning–based anomaly detection models were investigated to enable earlier awareness of production degradation and to support adaptive demand-side control before critical imbalance conditions occur. LR, RF, and XGBoost models were evaluated with respect to detection timing, control effectiveness, and robustness under fault-prone operating conditions. It should be noted that proactive detection timing and proactive control effectiveness are distinct concepts; early anomaly detection does not necessarily imply effective preventive control.

In this sense, the evaluated scenarios provide a controlled comparison of the main control configurations considered in this study. The no-balancing and reactive-balancing cases isolate the effect of delayed intervention, whereas the LR-, RF-, and XGBoost-based proactive cases compare different combinations of detection timing and control magnitude estimation under the same production degradation trajectory. Therefore, the analysis does not only compare classification models, but also examines how different control decision outputs affect the ability of the framework to prevent production–consumption imbalance.

**Figure 7:**
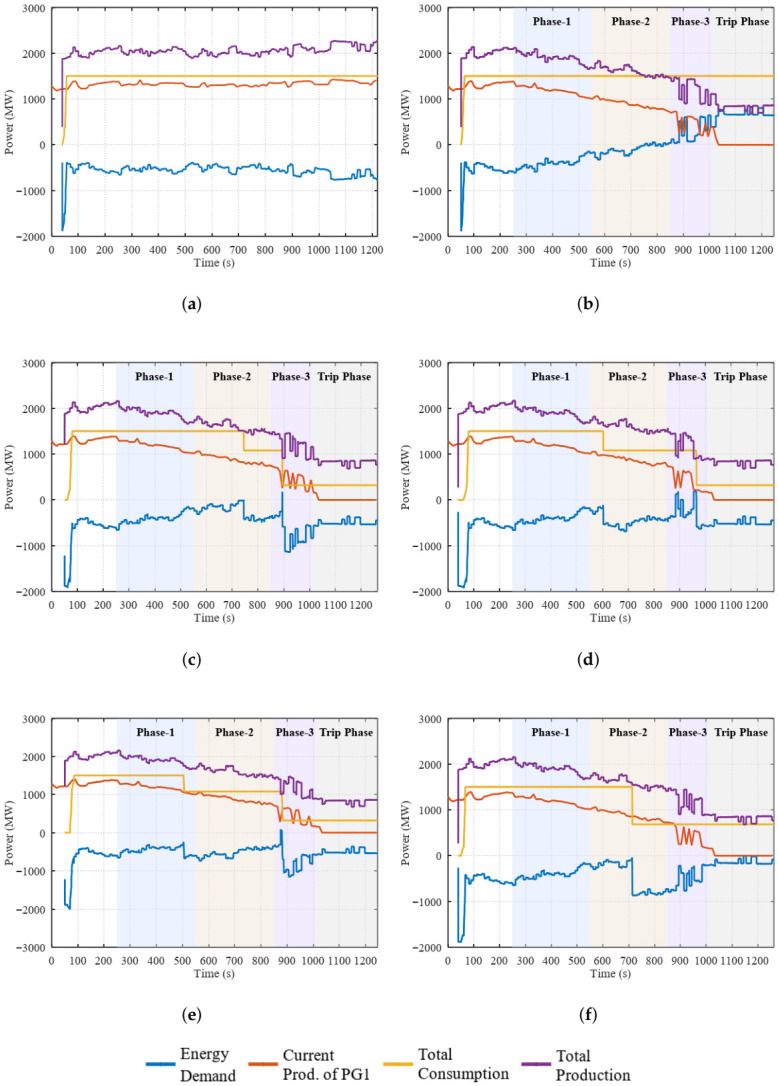
(**a**) No fault scenario (**b**) Fault scenario without load balancing (**c**) Fault scenario with reactive load balancing (**d**) Fault scenario with proactive load balancing using LR (**e**) Fault scenario with proactive load balancing using RF (**f**) Fault scenario with proactive load balancing using XGBoost.

Figure 7a illustrates the normal operation scenario, where electricity production and consumption remain balanced over time. As shown in the figure, the proposed framework does not generate false anomaly detections or trigger unnecessary control actions under stable operating conditions, confirming its robustness during fault-free grid operation.

Figure 7b presents a fault scenario without adaptive load balancing. In this case, a production drop occurs at the generation unit while total electricity consumption remains unchanged. This leads to a clear production–consumption imbalance and unstable system behavior, highlighting the necessity of adaptive demand-side control under production degradation events.

Figure 7c illustrates the fault scenario under a reactive load balancing strategy. Demand-side control actions are initiated only after total electricity consumption exceeds available production capacity. As a result, energy-saving measures are applied with a delay, during which the system operates under a temporary production–consumption imbalance. This delayed response demonstrates the limitations of reactive demand-side control mechanisms.

Following the reactive load balancing analysis, the LR–based approach under a proactive demand-side control strategy is evaluated, as shown in Figure 7d. The LR model detects production degradation during Phase 2 of the fault evolution and proactively triggers energy-saving commands before a severe imbalance emerges.

However, due to its linear modeling structure, LR fails to accurately estimate the magnitude of the required energy saving under progressively evolving and nonlinear degradation conditions. As the fault severity increases, the computed demand reduction becomes insufficient, leading to temporary production–consumption imbalance. This behavior explains the model’s low precision and high false positive rate, despite its early detection capability. From a control perspective, this results in insufficient and delayed corrective actions, limiting the practical usefulness of early detection alone. This limitation stems from the inherently linear decision boundary of LR, which restricts its ability to model nonlinear degradation trajectories and delayed compounding effects in production dynamics.

The RF–based approach demonstrates improved performance, as illustrated in Figure 7e. By capturing nonlinear feature interactions, the RF model detects production degradation at an earlier stage, typically during Phase 1, and provides a more accurate estimation of the required energy saving ratio compared to LR.

As a result, adaptive load control actions initiated by the RF model reduce the duration and severity of production–consumption imbalance. Nevertheless, under rapidly evolving fault conditions, the estimated demand reduction remains insufficient to fully stabilize the system. This behavior indicates that independent tree aggregation may limit the refinement of control magnitude estimation as the fault progressively evolves.

Finally, the XGBoost-based approach exhibits the strongest overall performance among the evaluated models, as shown in Figure 7f. Although XGBoost detects production degradation later than LR and RF when evaluated solely based on reactive detection timing, it consistently provides the most accurate estimation of the required energy saving ratio. The longer reactive detection delay observed for XGBoost does not indicate slower fault awareness; rather, it reflects that reactive intervention becomes less relevant because effective preventive control actions are already applied with accurate magnitude.

This precise control parameter estimation enables adaptive load control actions to maintain production–consumption balance continuously, preventing energy demand violations and ensuring stable system operation. These observations confirm that, in smart grid control applications, the value of an anomaly detector should be evaluated not only by how early it detects a fault, but by how effectively it enables corrective action.

Quantitative performance metrics corresponding to these observations are summarized in Table 4. For the XGBoost-based approach, proactive lead time represents the interval between the first preventive control action and the instant at which an energy demand violation would otherwise occur. While LR achieves perfect recall, it suffers from low precision and a high false positive rate. RF offers a more balanced detection performance but remains constrained in control accuracy. In contrast, XGBoost achieves the highest accuracy, precision, F1-score, and AUC-ROC, confirming its superior reliability and effectiveness for adaptive demand-side energy management. Accordingly, detection delay and lead time metrics are interpreted in conjunction with control effectiveness rather than as standalone indicators of model performance.

## 6. Conclusions

This study presented an AIoT-based adaptive energy management framework designed to support online adaptive demand-side control in smart grid environments under production degradation conditions. Unlike conventional approaches that rely on production forecasting or purely reactive load shedding strategies, the proposed framework focuses on detecting production drop anomalies at generation units and translating these anomalies into priority-aware and adaptive demand-side control actions.

The main contribution of the study is the integration of production-side anomaly awareness, control-oriented energy saving estimation, and distributed priority-aware load regulation within a single online AIoT framework. The proposed architecture also establishes a practical separation of authority and responsibility: the energy data center determines the required global energy saving ratio based on production-side anomaly awareness, while building-level gateways apply this objective locally according to priority policies defined by building managers or authorized users. This prevents the central system from embedding building-specific business rules and allows local operators to adapt load regulation to user preferences, occupancy, and operational constraints.

A key insight of this work is that triggering control actions solely based on energy demand violations is insufficient and potentially harmful, as it allows temporary production–consumption imbalance to occur and may introduce operational stress in real power systems. To address this limitation, machine learning–based anomaly detection models were employed to anticipate production degradation and enable preventive demand-side control strategies.

Comparative evaluation of LR, RF, and XGBoost models demonstrates that early anomaly detection alone does not guarantee effective system stabilization. Although LR and RF enable earlier, proactive fault awareness by detecting degradation during earlier fault phases, both models exhibit limitations in accurately estimating the required energy saving ratio under progressive and nonlinear fault conditions. In contrast, the XGBoost model, despite exhibiting a longer reactive detection delay, consistently provides precise control parameter estimation, enabling stable and uninterrupted grid operation.

These findings indicate that, in smart grid control applications, accurate estimation of the corrective control magnitude is at least as critical as early fault detection. Models that detect anomalies earlier but fail to compute sufficient control actions may still allow temporary imbalance, whereas models that provide accurate control magnitude estimation can achieve truly preventive system behavior. By integrating machine learning–based anomaly detection with IoT-enabled, priority-aware adaptive load control, the proposed framework offers a scalable, reliable, and practical solution for demand-side energy management in fault-prone smart grid environments.

The main limitation of the current study is that the framework is evaluated within a real data–driven simulation environment rather than a full grid-dynamic simulation or field deployment. Therefore, detailed grid-dynamic variables such as frequency and voltage deviations are not directly modeled. Since the EPİAŞ dataset does not include electromechanical grid variables such as frequency or voltage measurements, the present evaluation interprets operational robustness at the demand-side management level through production–consumption balance and prevention of energy demand violations. Detailed frequency- and voltage-based stability analysis is therefore left for future work with grid-dynamic simulation or field measurement data.

Future work will focus on validating the proposed approach through real-world deployments, extending the framework to accommodate diverse fault evolution patterns, and investigating cooperative and region-level control strategies to further enhance robustness, scalability, and operational resilience.

## Figures and Tables

**Table 1:** Comparison with recent AI-, IoT-, and AIoT-assisted smart grid studies.

Comparison Criterion	Wang et al. [28]	Liu et al. [29]	Singh et al. [30]	Khan et al. [31]	Proposed Framework
Main focus	Smart grid stability prediction	Power production anomaly detection	IoT-based adaptive resource allocation	Fog-based smart grid data analytics	**AIoT-based** **adaptive** **energy** **management**
AI/ML method	LR, RF, XGBoost, CatBoost, and other ML models	Neural normal stochastic process model	Deep learning and IoT-driven optimization	Fog-enabled secure data analytics	**LR, RF,** **and XGBoost**
Production-side anomaly/fault focus	No	Yes	No	No	**Yes**
Online/adaptive operation	Limited	Limited	Yes	Yes	**Yes**
Demand-side control	No	No	Yes	No	**Yes**
Priority-aware device-level control	No	No	No	No	**Yes**
Direct fault-to-control mapping	No	No	No	No	**Yes**

**Table 2:** Trade-off comparison of the selected machine learning models.

Model	Model Family	Interpretability	Computational Cost	Inference Latency	Predictive Capacity
LR	Linear classifier	High	Low	Low	Low–Medium
RF	Bagging ensemble	Medium	Medium–High	Medium	Medium–High
XGBoost	Boosting ensemble	Medium–Low	High	Medium–High	High

**Table 3:** Key Simulation Parameters.

Parameter	Value
Simulation duration	24 h
Number of IoT devices	>500
MQTT message interval	10 s
Number of buildings	5
Number of generation units	3

**Table 4:** Comparative Performance Evaluation of Machine Learning Models for Production Drop Anomaly Detection.

Metric	LR	RF	XGBoost
Accuracy (%)	28.0	85.6	99.4
Precision (%)	18.0	48.4	83.3
Recall (%)	100.0	40.0	100.0
F1-score (%)	30.0	43.8	90.9
Reactive Detection Delay (s)	355	260	570
Proactive Lead Time (s)	N/A	N/A	1.0
False Positive Rate (%)	85.3	6.9	0.66
AUC-ROC	0.54	0.76	0.999

Reactive detection delay is measured with respect to post-fault response, whereas proactive lead time indicates the anticipation interval prior to an energy demand violation.

## Data Availability

The electricity production data used in this study were obtained from the publicly accessible EPİAŞ Transparency Platform. The simulation data and implementation details generated during the study are available from the corresponding author upon reasonable request.
